# A Stochastic Characterization of Hydrogen Peroxide-Induced Regulated Cell Death in *Microcystis aeruginosa*

**DOI:** 10.3389/fmicb.2021.636157

**Published:** 2021-07-28

**Authors:** Leda Giannuzzi, Tomás Lombardo, Iván Juárez, Anabella Aguilera, Guillermo Blanco

**Affiliations:** ^1^Centro de Investigación y Desarrollo en Criotecnología de Alimentos, Consejo Nacional de Investigaciones Científicas y Tecnicas, Universidad Nacional de La Plata, La Plata, Argentina; ^2^Área de Toxicología General, Facultad de Ciencias Exactas, Universidad Nacional de La Plata, La Plata, Argentina; ^3^Laboratory of Immunotoxicology (LaITo), Facultad de Farmacia y Bioquímica, Instituto de Estudios de la Inmunidad Humoral Dr. Ricardo A. Margni (IDEHU) UBA-CONICET, Universidad de Buenos Aires, Buenos Aires, Argentina; ^4^Centre for Ecology and Evolution in Microbial Model Systems (EEMiS), Linnaeus University, Kalmar, Sweden

**Keywords:** cyanobacteria, *Microcystis*, cell death, regulated cell death, binary logistic function, EC_50_

## Abstract

Regulated cell death (RCD) encompasses the activation of cellular pathways that initiate and execute a self-dismissal process. RCD occur over a range of stressors doses that overcome pro-survival cellular pathways, while higher doses cause excessive damage leading to passive accidental cell death (ACD). Hydrogen peroxide (HP) has been proposed as a potential tool to control harmful cyanobacterial blooms, given its capacity to remove cyanobacterial cells and oxidize cyanotoxins. HP is a source of hydroxyl radicals and is expected to induce RCD only within a limited range of concentrations. This property makes this compound very useful to better understand stress-driven RCD. In this work, we analyzed cell death in microcystin-producing *Microcystis aeruginosa* by means of a stochastic dose response model using a wide range of HP concentrations (0, 0.29, 1.76, 3.67, 7.35, 14.70, and 29.5 mM). We used flow cytometry and unsupervised classification to study cell viability and characterize transitional cell death phenotypes after exposing cells to HP for 48 and 72 h. Non-linear regression was used to fit experimental data to a logistic cumulative distribution function (cdf) and calculate the half maximal effective concentration (EC_50_). The EC_50_ of *M. aeruginosa* exposed to HP were 3.77 ± 0.26 mM and 4.26 ± 0.22 mM at 48 and 72 h, respectively. The derivative of cdf (probability density function; pdf) provided theoretical and practical demonstration that EC_50_ is the minimal dose required to cause RCD in 50% of cells, therefore maximizing the probability of RCD occurrence. 1.76 mM HP lead to an antioxidant stress response characterized by increased reactive oxygen species (ROS) levels and HP decomposition activity. The exposure of 3.67 mM HP induced a dose-related transition in cell death phenotype, and produced several morphological changes (a less dense stroma, distortion of the cell membrane, partial disintegration of thylakoids, extensive cytoplasmic vacuolation and highly condensed chromatin). The EC_50_ and the stochastic cdf and pdf together with the multidimensional transitional phenotypic analysis of single cells contribute to further characterize cell death pathways in cyanobacteria.

## Introduction

Cyanobacteria are a group of organisms with great ecological and economic importance (Dittmann and Wiegand, 2006). The increase in cyanobacterial blooms, linked to climate change and the eutrophication of water bodies, is a worldwide concern (Vasconcelos, 2015). Although cyanobacterial blooms are recognized as a significant health risk, only few of the known Cyanobacteria produce recognized toxins, including hepatotoxins such as microcystins (MCs). The conditions that promote their massive proliferations have been extensively studied, but considerably less is known about mechanisms and molecular machinery of cell death in cyanobacteria.

*Microcystis aeruginosa* is one of the most frequently studied cyanobacteria, due to its wide world distribution, and its well-recognized toxicity mediated by the production of hazardous microcystins (MCs) (Sivonen and Jones, 1999; Harke et al., 2016). Cell death in *M. aeruginosa* is critical for understanding harmful blooms and their responses to environmental abiotic and biotic adversities. Contrasting the paucity of studies in cyanobacteria, a large amount of knowledge has been accumulated about cell death in eukaryotes in the last decades. The main consensus is that Accidental Cell Death (ACD), which is unpreventable and is caused by intense damage to which the cell cannot respond, can be distinguished from Regulated Cell Death (RCD), an active process that relies on the intracellular molecular machinery and can therefore be modulated pharmacologically or genetically (Galluzzi et al., 2015). RCD can be activated in the context of physiological processes, such as post-embryonic development and ontogeny of the immune system (Taylor et al., 2008; Fuchs and Steller, 2011), and in that particular case it is designated as programmed cell death. On the other hand, RCD also occurs in the context of adaptation to stress, when responses to perturbations of the intracellular or extracellular microenvironment fail, and therefore it would constitute an ultimate attempt to maintain homeostasis (Galluzzi et al., 2018). It is important to emphasize that this terminology follows the conceptual framework and recommendations of the nomenclature committee on cell death (NCCD) (Galluzzi et al., 2018). A similar approach has been recently introduced for studies on cell death in cyanobacteria, including recommendations to characterize of RCD pathways by combining molecular, biochemical, and morphologic aspects following the directions of the (NCCD) (Aguilera et al., 2021). Therefore, we will hereon refer to RCD in all cases where a stressor triggers an active form of cell death regardless the specific cellular pathway involved (i.e., ferroptosis, pyroptosis, apoptosis, necroptosis, etc.) and whether these pathways are characterized or not.

Compounds that produce hydroxyl radicals seem promising for treating cyanobacterial blooms, because they have significantly higher toxicity to cyanobacteria than to green algae (Drábková et al., 2007). In particular, the use of hydrogen peroxide (HP) has been proposed as a potential tool to control harmful cyanobacterial blooms, given its capacity to induce cell death and oxidize cyanotoxins. Several studies report the potential of HP for removing *Microcystis* and MCs in different environments (Cornish et al., 2000; Drábková et al., 2007; Barrington and Ghadouani, 2008; Matthijs et al., 2012).

In *M. aeruginosa*, HP treatments lead to biochemical and morphological changes such as condensation of chromatin, cytoskeletal rearrangements with cell membrane disruption (blebbing), activation of caspases-like activities, and degradation of DNA to small fragments (Ding et al., 2012; Zhou et al., 2018) resembling RCD subroutines described in animals. Interestingly, HP treatments in *M. aeruginosa* are associated with a reduced release of toxins into the surrounding medium at moderate doses (0.29 mM) (Zhou et al., 2020).

Considering that RCD induced by harmful agents such as HP occurs within a limited range of doses, the characterization of cell death as a function of HP concentration is critical for the study of cyanobacterial death, and for the identification of genetic pathways involved in RCD and the observation of biochemical and morphological hallmarks. High-throughput single-cell methods such as flow cytometry involve the screening of thousands of individual cells that can be scored as dead or alive with great confidence, at several HP dose levels. Therefore, they allow the use of stochastic dose-response models such as the binary logistic function.

Using these models, parameters such as the effective dose that causes cytotoxicity in 50% of cells (EC_50_) can provide a biologically relevant measure for the assessment of active cell death. RCD is considered to be initiated once the several concurrent cellular pathways that attempt to restore homeostasis and preserve cell survival are overcome by the extent of cell damage inflicted. This conceptual point of no return can be translated in probabilistic terms to the EC_50_ dose in a population. At the EC_50_ concentration half of the cell population will be committed to cell death and the other half will still be alive due to pro-survival homoeostatic cellular pathways.

In this study, we analyze cell death in *M. aeruginosa* at 48 and 72h as a stochastic function of HP concentration (probabilistic dose response curve) aiming to determine the range within which RCD most probably occurs. We explore the correspondence between HP concentration and RCD-associated multidimensional phenotypic changes assessed by flow cytometry, together with morphological and biochemical features of RCD.

## Materials and Methods

### Culture Conditions and Experimental Assays

The experiments were performed with non-axenic cultures of [D-Leu^1^] MC-LR producer *Microcystis aeruginosa* strain CAAT 2005-3, isolated from Buenos Aires, Argentina (Rosso et al., 2014; Giannuzzi et al., 2016). Cells were grown in batch cultures in BG-11 media (Rippka et al., 1979) at 30 μE m^–2^ s^–1^ under 14:10 h light:dark photocycle at 26°C. Cultures in the exponential phase (10^6^ cells⋅mL^–1^; Chl-*a* 2,500 μg.L^–1^) were used for treatments with different concentrations of HP in a batch mode.

A stock solution of HP (3% w/w) was prepared from a commercial 30% (w/w) solution (Fluka) via dilution with Milli-Q water (Millipore, CA, United States) and stored at 4°C after filter sterilization. A stock solution of HP was used to make a calibration curve (5–1,000 mg L^–1^ HP). HP concentration was verified using potassium permanganate titration and then added to 50 ml of *M. aeruginosa* culture to achieve 10 mg.L^–1^ (0.29 mM), 60 mg.L^–1^ (1.76 mM), 125 mg.L^–1^ (3.67 mM), 250 mg.L^–1^ (7.35 mM), 500 mg.L^–1^ (14.70 mM), and 1,000 mg.L^–1^ (29.40 mM). Treatments were conducted in duplicates. Cultures of *M. aeruginosa* without the addition of HP were used as control.

The control and treated cultures were placed in a controlled environment growth chamber (Ingelab I-292PF) at 26°C under light conditions 30 μE m^–2^ s^–1^ under 14:10 h light:dark photocycle.

Samples for determination of reactive oxygen species (ROS) and measurement of HP decomposition activity were analyzed at 0 and 24 h after HP addition for doses close to EC_50_ (0, 1.76, 3.67, and 7.35 mM) (see below).

The possibility of re-growth of the culture after HP exposure was also evaluated. To this end, an equal volume of BG11 medium was added to each treated culture at the end of the experiment (72 h), and incubated under the same conditions for 15 days and then their appearance was observed. HP degradation was measured in triplicate at 24, 48, and 72 h by Quantofix test sticks (Machereye-Merck, Darmstadt, Germany) (Matthijs et al., 2012). According to previous experiments, we refine the measurements by making photographs of each test stick and subsequent comparison to our own calibration series (Juárez et al., 2020). HP degradation was tested in BG11 medium without cells as a control.

### Evaluation of Cell Viability by Flow Cytometry and N-Dimensional Analysis

Cell viability was assessed by flow cytometry using a Partec PAS III cytometer equipped with a blue 488 nm argon laser and a red 640 nm solid state laser as excitation sources. Fluorescence from cyanobacterial chlorophyll (Chl) was detected with a 660 nm band pass emission filter (FL3) after blue light excitation. Phycoyanin cyanobacterial fluorescence (PC) was detected with a 660 nm band pass filter (FL4) after red light excitation. Forward light scatter (FSC) and side light scatter (SSC) were both detected from 488 nm blue light through photomultipliers. SSC was used as an indicator of internal complexity (e.g., granular content, cytoskeletal rearrangements such as distortion of cell membrane and cell wall, increased number of vacuoles and the presence many other undetermined small intracellular constituents).

Viable *M. aeruginosa* cells were identified by FSC and SSC, together with intrinsic fluorescence of Chl and PC. SYTOX Green was used to assess non-viable cells as it only penetrates compromised membranes characteristic of dead cells. Cell suspensions were stained with SYTOX Green at a final concentration of 1 μM and incubated for 10 min protected from light. Non-viable cells were identified by green fluorescence emission at 520 nm (FL1). Therefore, using this approach, we considered five parameters (FSC, SSC, SYTOX Green, Chl, and PC) to evaluate the fraction of dead and alive *M. aeruginosa* cells at each time-point after treatment (48 and 72 h of HP exposure). Manual analysis of flow cytometry data was conducted with Flomax 2.7 software.

Dimensionality reduction and visualization of the five-dimensional space (FSC, SSC, SYTOX Green, Chl, and PC) was conducted following uniform manifold approximation and projection (UMAP) (Becht et al., 2018; McInnes et al., 2018) and t-distributed stochastic neighbor embedding (t-SNE) algorithms (van der Maaten and Hinton, 2008; Belkina et al., 2019). Flow Cytometry Standard (FCS) files obtained from control and HP-treated samples were concatenated into a single file where the sample ID tag was used for tracking the HP dose applied to each cell. Plots were produced with UMAP and tSNE packages from R-Bioconductor and through the plugins that access these R packages in FlowJo 10.5 software. Unsupervised classification was conducted with SPADE 3.0 using a free version for academic use (available at^1^) (Qiu et al., 2011; Qiu, 2012), and with the R package flowSOM (Van Gassen et al., 2015) either directly or through the plugin access available from FlowJo 10.5 software.

### Measurement of Reactive Species (DCFH-DA Oxidation Rate)

Intracellular ROS was measured using 2’, 7’-dichlorodihydrofluorescein diacetate (DCFH-DA). Six milliliter samples were filtered through a glass fiber filter (GF/F) and washed twice with PBS. Filters were incubated at 27°C in the dark for 30 min in 1,950 μL of 40 mM Tris-HCl buffer (pH 7.0) and 50 μL of 0.5 μM of DCFH-DA solution (Malanga et al., 2001). After centrifugation at 5,000 g for 15 min, the fluorescence of the supernatant was monitored in a microplate reader (Beckman counter DTX 880, Multimode Detectors) with excitation (λ_*ex*_) at 498 nm and emission (λ_*em*_) at 525 nm. In all cases, parallel blank controls were included.

### HP Decomposition Activity

The measurement of HP decomposition was performed by UV spectrophotometric method. HP decomposition can be followed directly by its consumption at 240 nm during 5 min (Beutler, 1984). One unit of HP decomposition was defined as the amount of enzyme catalyzing the elimination of 1 mM HP per minute. This assay is typically used to indirectly determine catalase activity in *M. aeruginosa* (Tytler et al., 1984; Giannuzzi et al., 2016; Du et al., 2017; Hernando et al., 2018; Ross et al., 2019; Li et al., 2020). In this work we refer the results to as measurement of HP decomposition instead of catalase activity since it is not clear which gene products are responsible for HP decomposition in *M. aeruginosa* (e.g., peroxiredoxins, or other proteins containing catalase-like domains). In addition, co-occurring heterotrophic bacteria in the non-axenic culture could also be responsible for HP decomposition.

### Transmission Electronic Microscopic TEM

Morphological changes in cells exposed to HP were assessed by TEM at 72 h of exposure. 10 ml of cultures were collected by centrifugation (1,500 rpm for 10 min at 4°C) and fixed with 2% glutaraldehyde in phosphate buffer (pH 7.2–7.4) for 2 h at 4°C. A secondary fixation was performed with 1% osmium tetroxide for 1 h at 4°C. After graded alcohols dehydration, samples were embedded in Epoxy resin. Sections were cut, stained with uranyl acetate and lead citrate and examined with a JEM 1200 EX II transmission electron microscope (JEOL Ltd., Tokyo, Japan) and photographed with an Erlangshen ES1000W camera, Model 785 (Gatan Inc., Pleasanton, CA, United States) of the Central Service of Electronic Microscopy of the Faculty of Veterinary Sciences, UNLP.

### Light and Fluorescence Microscopy

Slide preparations were observed in an Olympus BX-51 fluorescence microscope equipped with a 100 Watt mercury lamp, a halogen lamp for transmitted light, U-plan fluorite objectives and three fluorescence filter cubes (U-MWB2 and U-MWG2 for blue and green excitation light, respectively). All images were acquired with a digital Q-Color 3 Olympus camera and Image-Pro Plus 6.0 software (Media Cybernetics, United States). Image processing was performed with Image-Pro Plus 6.0. Monochrome layers from RGB images were digitally separated, pseudocolored, and segmented with ImagePro Plus 6.0. For deconvolution images, improvement of edge detection was obtained with fast Fourier transformation (FFT) and Laplace filter. Image merging was also done with Image Pro Plus 6.0.

### [D-Leu]^1^MC-LR Quantification

The content of [D-Leu]^1^MC-LR was evaluated at time zero and at 72 h of HP exposure at EC_50_. Similarly, [D-Leu]^1^MC-LR production were performed in control samples. 10 ml of culture were submitted to ultrasonication for 30 min (Omni Ruptor 400) and then centrifuged (15 min at 5,000 rpm) to eliminate cell debris. The supernatant was passed through previously conditioned Sep-Pak C18 cartridges (Waters) (10 mL 100% methanol, 50 mL 100% distilled water). The filtrate with MCs was eluted with 80% methanol (Barco et al., 2002). The quantitative analysis of MCs was performed by HPLC/MS (Shimadzu LCMS-2020) to determine the principal component of [D-Leu^1^] MC-LR toxins (m/z 520) using a C18 column (Hyperprep HS, 5-μm pore, 250 mm 10 mm) according to Giannuzzi et al. (2016). Standard of MC-LR was purchased from Sigma (St. Louis, MO, United States). [D-Leu]^1^ MC-LR was expressed as μg.L^–1^.

### Data Analyses

In the toxicity tests, experiments were conducted in duplicate and the mean and standard deviation were calculated. The data were tested for homogeneity and normality of variance before analysis. The dose expected to produce cell death in 50% of treated cells was defined as EC_50_. The EC_50_ value was calculated from the parameters of a logistic cumulative probability function using non-linear regression methods of analysis with the statistical program SPSS 10.0.

Tests for significance of differences between the EC_50_ values at 24 and 72 h were determined using the Analysis of variance (ANOVA) and comparison tests according to the Fisher significant differences table (least significant difference) were applied with significance levels of 0.05. For [D-Leu]^1^MC-LR, ROS and measurement of HP decomposition, data were expressed as mean ± standard error (SE) and analyzed using a ANOVA. Differences were considered to be significant at *p* < 0.05 (^∗^) and p < 0.01 (^∗∗^).

## Results

### Appearance of *M. aeruginosa* Culture Treated With HP

**FIGURE 1:**
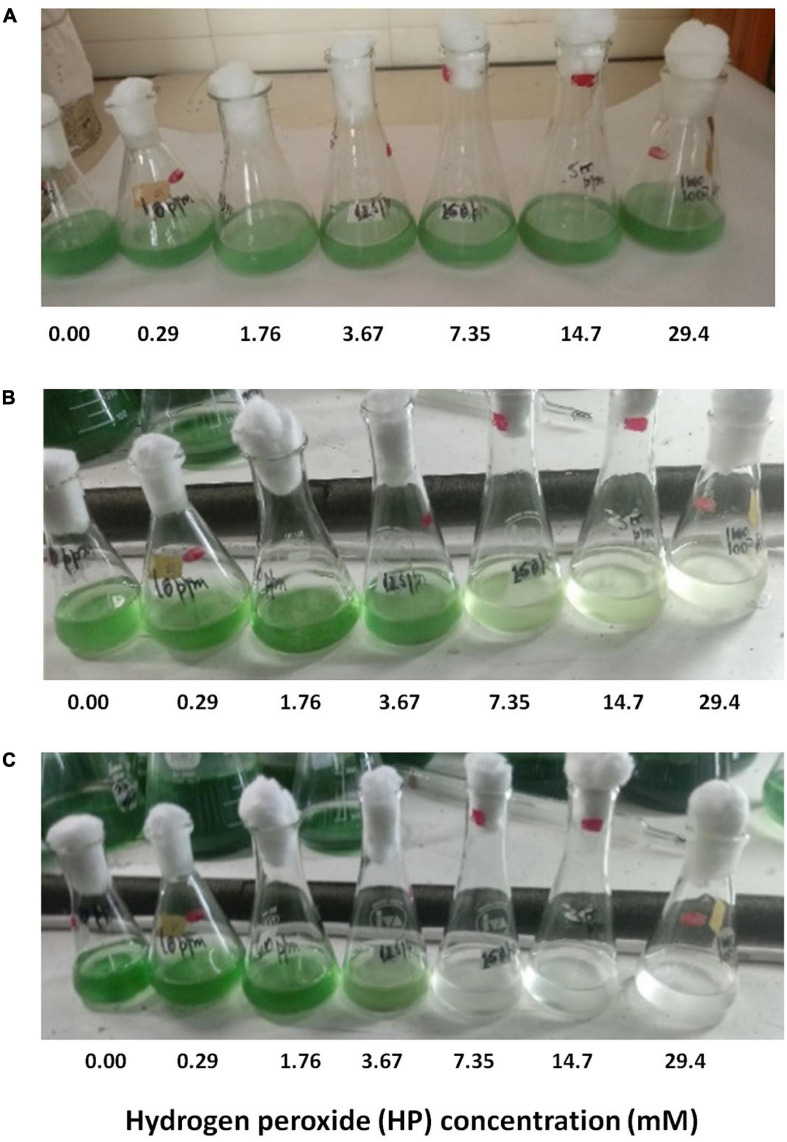
Effect of addition of HP on the appearance of *M. aeruginosa* culture after 0, 48, and 72 h of HP treatment [**(A–C)**, respectively].

Culture discoloration became apparent with increasing concentrations of HP. The translucent appearance of *M. aeruginosa* cultures was observed at 48 h, and became more noticeable after 72 h in samples containing higher HP concentrations (Figure 1).

### Identification of Dead Cells by Flow Cytometry

**FIGURE 2:**
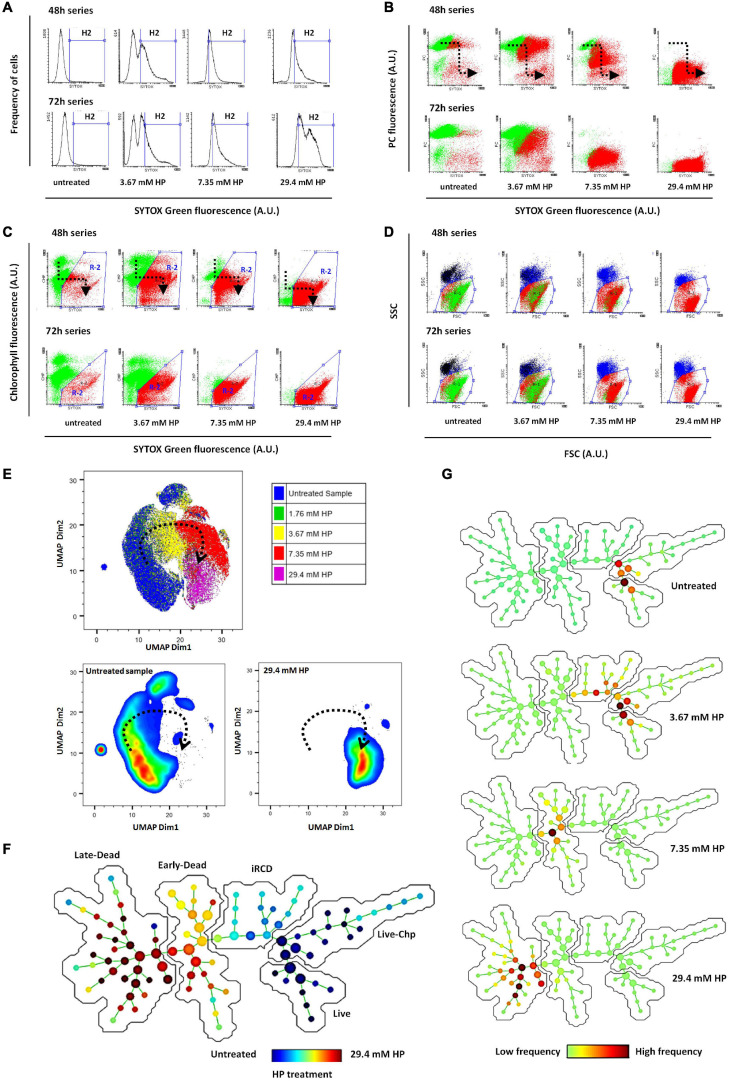
**(A)** Frequency distribution of SYTOX Green fluorescence among HP-treated cells at 48 and 72 h (Control and three representative HP doses). Dead cells were identified as the fraction of the cell population within the H2 region of histograms. **(B)** Bidimensional distribution (correlation plot) of SYTOX Green fluorescence vs. Phycocyanin (PC) fluorescence. Loss of selective membrane permeability and labeling of DNA by SYTOX Green occurred prior to loss of PC fluorescence. The black dotted arrow indicates the direction of progressive fluorescence changes as the concentration of HP is increased (“death pathway”). Green cluster corresponds to live cells and red cluster corresponds to dead cells. **(C)** Bidimensional distribution of SYTOX Green fluorescence vs. Chlorophyll (Chl) fluorescence. Loss of membrane selective permeability and labeling of DNA by SYTOX Green, occurred after partial loss of Chl fluorescence [Black arrow indicates “death pathway” as noted in **(B)**]. R2 corresponds to the subset of dead cells as indicated by increased SYTOX Green fluorescence and decreased Chl fluorescence. Green cluster: live cells; red cluster: dead cells. **(D)** Bidimensional distribution (correlation plot) of forward scattered light (FSC) vs. side scattered light (SSC). Green and red clusters correspond to viable and dead cells, respectively, as classified from R2 illustrated in **(C)**. **(E)** UMAP bidimensional plot. The five-dimensional location of cells according to FSC, SSC, SYTOX Green, Chp, and PC values was visualized in 2D by UMAP dimensionality reduction. The treatment applied to each cell is indicated by color code. Lower plots show only live cells on the left and only dead cells at highest HP dose on the right (dead cells control). The dotted arrow indicates the transition of the five-dimensional phenotype as HP was increased. The complete transitional phenotype series is shown in Supplementary Figure 1. Comparable results obtained using t-SNE visualization are presented in Supplementary Figure 2. **(F)** Tree-like structure produced by SPADE algorithm showing 100 nodes consisting of very similar cells. Node size is proportional to the amount of cells in each node. Color code indicates the relative enrichment of nodes according to the HP treatment applied to cells. The average values of each of five parameters in every node are shown as heatmap SPADE graphs in Supplementary Figure 3. Phenotypic transition and exploration of node parameter values allowed subsequent metaclustering in three dead groups and two live groups (iRCD, initial regulated cell death metacluster; Live Chp, live cells with high Chp content; see Supplementary Figure 3 for node parameter values). Distance along the path between any two nodes is proportional to their phenotypic dissimilarity. **(G)** SPADE graphs of individual samples, used to identify their location along the phenotypic transitional path. Color code indicates the enrichment of each particular sample among the 100 nodes (frequency of cells from a given sample relative to the frequency from all samples).

Phycocyanin (PC) and chlorophyll (Chl) fluorescence, forward light dispersion (FSC) and side light dispersion (SSC) were considered in addition to SYTOX Green to classify cells as dead or alive. Dead cells were identified by green fluorescence of SYTOX Green. The fraction of SYTOX Green-positive cells increased dose-dependently at 48 and 72 h (Figure 2A). At the same time, the fluorescence detected from phycocyanin decreased dose-dependently but only in SYTOX Green positive cells, indicating that degradation of PC occurred later than the membrane damage (Figure 2B). By contrast, Chl fluorescence appeared to decrease earlier than membrane damage, indicating that this event could take place during the initial phase of active cell death (Figure 2C). The analysis of light dispersion parameters showed that active cell death was associated with a dose dependent decrease in both FSC and SSC. This is illustrated by the progression of the red cluster shown in Figure 2D. The overall pattern of transition from live to dead phenotype was similar in cells treated during 48 and 72 h.

#### RCD-Related Transition of Five-Dimensional Phenotypic Signature Was Induced by Increasing HP Doses

To confirm the transitional phenotype identified by conventional manual analysis, we conducted a complementary analysis using dimensionality reduction and clustering algorithms. Using five parameters to determine cell death not only increased the confidence of scoring, but also provided a phenotypic signature that enabled the classification of the entire population according to their similarity. Therefore, the occurrence of RCD should be reflected as a transition of this five-dimensional phenotypic signature (FSC, SSC, SYTOX Green, Chl, and PC) in accordance with the HP dose applied.

We first visualized the entire dataset of samples treated with increasing HP doses in a five dimensional space, through the two-dimensional reduction provided by the UMAP algorithm (Figure 2E). In UMAP plot, live cells from control samples appeared separated from dead cells of samples treated with the highest HP dose (29.4 mM) (Figure 2E). Cells from samples treated with intermediate doses such as 3.67 and 7.35 mM HP appeared in locations in between these two live and dead control extremes. Cells from samples treated with low doses such as 1.76 mM HP had locations overlaid to cells from untreated samples (Figure 2E). In addition, by plotting a third parameter as a heatmap, the UMAP graph allowed tracking transitional changes in PC, Chl, SYTOX Green, FSC and SSC in accordance with HP dose (Supplementary Figure 1). A similar result was obtained with visualization of the five-dimensional space using the t-SNE algorithm although the transitional phenotype was better visualized with UMAP (Supplementary Figure 2).

In a complementary approach, we used the “spanning-tree progression analysis of density-normalized events” (SPADE) clustering algorithm to identify 100 small groups of very similar cells called nodes, and further represented these nodes in a branched-tree graph in two dimensions (Figure 2F). The distance between two nodes in the branched-tree is proportional to their phenotypic dissimilarity. As expected, nodes corresponding to dead cells treated with 29.4 mM HP appeared well apart from nodes corresponding to untreated live cells, and the branched tree allowed the representation of the phenotypic transition from live cells to dead cells (Figures 2F,G).

SPADE allows a second step of manual clustering where the numerous small nodes are grouped into larger clusters (metaclusters) based on partially shared phenotypes. Using this approach, we identified three metaclusters of dead cells that we called iRCD, early-dead and late-dead cells (Figure 2F and Supplementary Figure 3). We assumed that the iRCD metacluster corresponded to the phenotype during the earliest step in the initiation of RCD, while early-dead and late-dead metaclusters corresponded to the more advanced phenotypes, based on the progression shown in the SPADE tree representation. In addition, we identified two metaclusters of live cells based on Chl content (Figure 2F and Supplementary Figure 3). We obtained a similar arrangement of transitional phenotypes along a gradient of HP dose in a branched-tree representation with 144 nodes using the flowSOM clustering algorithm (Supplementary Figure 4).

#### Changes Induced by HP in Single and Combined Flow Cytometry Parameters

**FIGURE 3:**
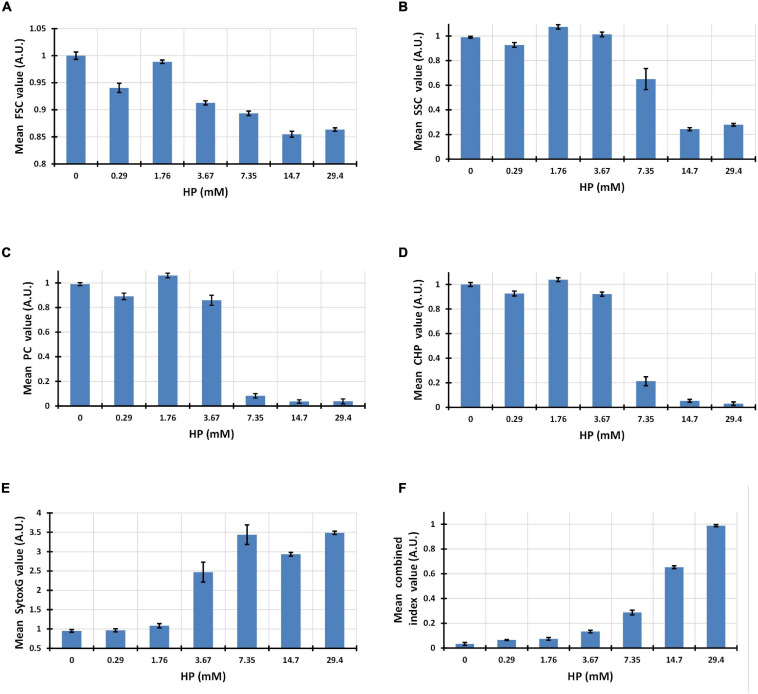
Bar graphs showing decrease in forward light scatter (FSC) and side light scatter (SSC) with increasing HP concentrations **(A,B)**. Progression of PC and Chl mean fluorescence values with increasing HP concentrations **(C,D)**. Increase in the mean fluorescence intensity of SYTOX Green after exposure to increasing HP concentrations **(E)**. Progressive increase of a virtual combined parameter defined as (2*SYTOX Green)/(PC + Chl) and measured at single cell level **(F)**.

Although the five-dimensional phenotype showed a smooth transition in clustering analysis, each parameter had its own change pattern when observed individually. Forward light dispersion is correlated to individual cell size. An overall decrease in cell size was observed in FSC along the entire HP dose range, achieving 15% less of basal value above concentration of 14.7 mM HP (Figure 3A). Side scatter light dispersion, which correlates with internal complexity, was reduced up to 80% over basal values and maximal decrease was achieved above 14.7 mM HP (Figure 3B).

PC fluorescence as indicated by mean fluorescence intensity of 100,000 cells, was found to be close to basal levels up to 3.67 mM HP, decreasing abruptly to less than 10% of basal values above 7.35 mM HP (Figure 3C).

Similarly, Chl fluorescence fluctuated around basal level values up to 3.67 mM HP, but decreased abruptly to less than 20% of basal levels at 7.35 mM HP and higher (Figure 3D).

Fluorescence from SYTOX Green indicated labeling of DNA after damage of cyanobacteria cell wall and cell membrane. Almost no increase of DNA labeling was observed up to 1.76 mM, but was abruptly increased to about 2.5 times its basal level at 3.67 mM HP, and achieved maximal levels at 7.35 mM HP (Figure 3E).

Flow cytometry data processing allows the measurements of virtual parameters in individual cells created by combination of several single parameters. We used this feature to observe a combined parameter of SYTOX Green, Chl, and PC. The fluorescence intensity of this virtual combined-parameter [Index values = 2^∗^SG/(Chl + PC)] showed a smooth increase as a function of HP dose, achieving a maximal value only at the highest dose of 29.4 mM HP (Figure 3F).

#### Probabilistic Modeling of HP-Induced Cyanobacterial Cell Death

By assessing FSC, SSC, PC, Chl, and SYTOX Green we determined whether each cell was alive or dead in a 100,000 sample at each HP dose point. This kind of binary outcome that follows a simple Bernoulli distribution as a function of a continuous variable is easily modeled by a logistic function:

(1) $$c\,p\,f_{d\,e\,a\,d}\,\left(x\right)=\frac{e^{u\,\left(x\right)}}{1+e^{u\,\left(x\right)}}$$

Where,

(2) u⁢(x)=Aln⁢(x)+B

**FIGURE 4:**
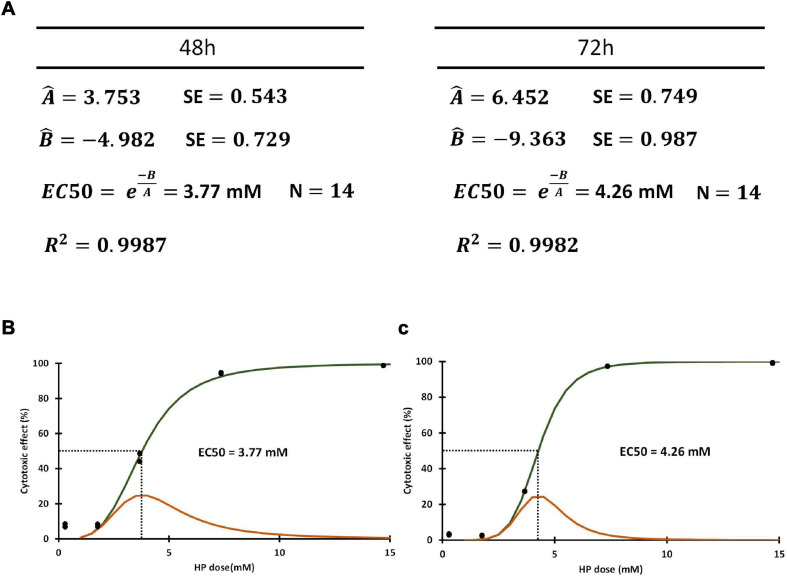
**(A)** Estimators obtained for slope (Â) and intercept (B̂) through non-linear regression and used to compute EC50. **(B)** Cumulative probability function obtained using estimators for **(A,B)** (cdf_dead) in green line. The pdf function (pdf_dead) obtained as the first derivative of cdf_dead in orange line. Both functions correspond to 48 h HP exposure. Dotted line corresponds to EC50 value. **(C)** The cdf_dead and pdf_dead functions (green and orange line, respectively) corresponding to 72 h HP exposure. Dotted line corresponds to EC50 value.

The independent variable x corresponds to HP concentration, and the function will predict the cumulative probability of cell death at any given dose level (cpf_dead). The logistic function is amenable to non-linear regression in order to obtain estimators of parameters A and B. The results that we obtained for our 48 and 72 h assays are shown in Figure 4A.

In addition, the probability that a cell will die at any particular HP concentration x can be obtained by the derivative of Equation (1), which represents a probability density function (pdf_dead):

(3) $$p\,d\,f_{d\,e\,a\,d\,\left(x\right)}=\frac{\partial }{\partial x}\,\left[\frac{e^{u\,\left(x\right)}}{1+e^{u\,\left(x\right)}}\right]=\frac{e^{u\,\left(x\right)}}{{\left(1+e^{u\,\left(x\right)}\right)}^{2}}$$

Where x is HP concentration and ì(x) is as defined by Equation (2).

Interestingly for cpf_dead(x) = 0.5, x = −B/A, therefore allowing the straightforward estimation of the HP concentration where the probabilities of death and survival are equal (EC_50_). The estimators of EC_50_ at 48 and 72 h were 3.77 ± 0.26 mM and 4.26 ± 0.22 mM, respectively (Figure 4A).

The plot of the estimated cdf_dead(x) and pdf_dead(x) functions at 48 and 72 h are shown in Figures 4B,C. No significant differences were found between EC_50_ at 48 and EC_50_ at 72 h (*p* = 0.487).

An important observation from the function pdf_dead(x) is that EC_50_ is the most frequent HP dose required to elicit cell death in the entire population. The left and right tails of this distribution function show the frequency of cells requiring more than EC_50_ HP dose (resistant group), and of those cells requiring less than EC_50_ HP dose to be killed (susceptible group). The longer right tail and skewed distribution of pdf_dead(x) observed at 48 h (Figure 4B) as compared to 72 h (Figure 4C), indicated that there was a larger group of resistant cells in the former group as compared to the latter.

Since the minimal dose eliciting death in a given cell most likely induces active cell death, in any alternative form (RCD as apoptosis, necroptosis, ferroptosis, etc.), the function pdf_dead(x) represents also a function predicting the theoretical frequency of RCD occurrence for any given HP dose value. However, in a real scenario, the observation of RCD morphological and biochemical features occurring at EC_50_ will also include those cases that require less than EC_50_ to initiate death, and only cumulative figures can be observed in practice.

### Biochemical and Morphological Features of RCD in HP-Treated Cyanobacteria

Flow cytometry allows fluorescent and refracted light single cell multi-parametric assessment but resigns imaging for the sake of massive quantification. Thus, morphology assessment demands alternative methods. However, there is a perfect correspondence between multi-parametric flow cytometry assessment and fluorescence or light microscopy.

**FIGURE 5:**
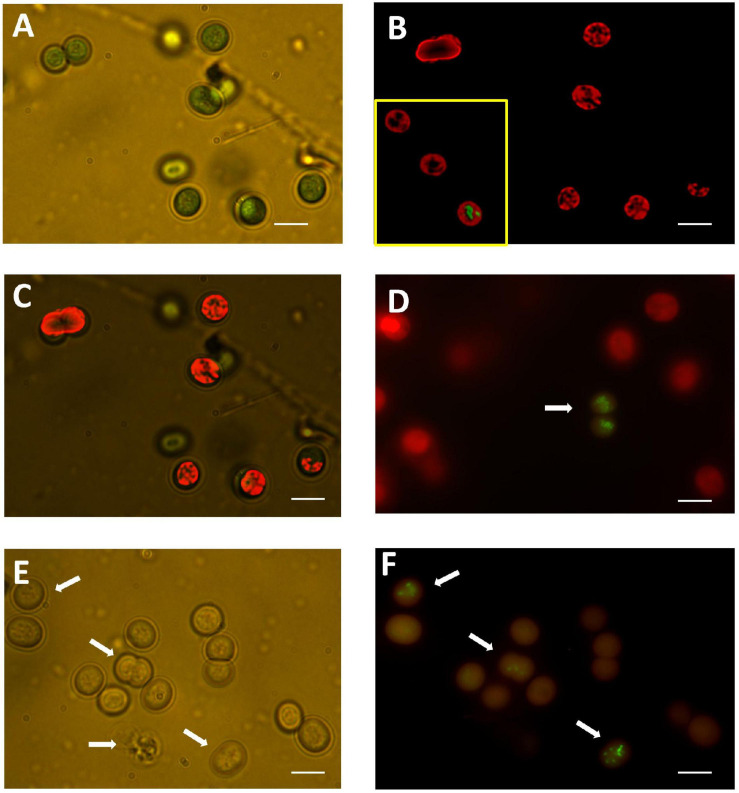
**(A)** Untreated sample observed with transmitted light at 1,000 x with immersion oil. Green color corresponds to light absorbed from thylakoid pigments. **(B)** A de-convoluted image of the same field observed with fluorescence microscopy to appreciate the distribution of fluorescence emitted from PC and Chl. The inset shows the appearance of the DNA in a rare cell with membrane damage as indicated by SYTOX Green staining (taken from another field of the untreated sample). **(C)** Merged image from **(A,B)** to appreciate the correspondence of light and fluorescence imaging. **(D)** A sample treated with 0.29 mM HP at 72 h of exposure showed barely detectable dead cells labeled with SYTOX Green (arrow). Labeling of DNA with SYTOX Green was associated to dim fluorescence from PC and Chl. Most cells treated with 0.29 mM HP retained the PC and Chl red fluorescence. **(E)** A sample treated with 7.35 mM HP at 72 h of exposure observed with transmitted light to show the disappearance of red color from thylakoid pigments. There was variable disruption of intracellular content (arrows). **(F)** Fluorescence image of the same sample in **(E)**. DNA labeling with SYTOX Green indicated loss of membrane selective permeability. Condensed and fragmented image of DNA with variable degrees of disintegration was in all cases associated with marked reduction of red fluorescence from Chl and PC (arrows). In all images bar size = 10 μm.

The normal appearance of unstained *M. aeruginosa* under light microscopy is shown in Figure 5A. The strong absorption of light by PC and Chl at higher wavelengths causes their normal light green color appearance in transmitted light microscopy (Figure 5A). The simultaneous assessment of fluorescence confirmed the intracellular distribution of PC and Chl in untreated cultures (Figures 5B,C).

As shown previously in Figures 3C,D, a dose of 0.29 mM HP at 72 h of exposure did not affect the fluorescence of either PC or Chl. This was corroborated by fluorescence microscopy (Figure 5D). The number of SYTOX Green-positive cells was low at 0.29 mM, but the few cells detected showed a clear condensation of DNA staining (Figure 5D). By contrast, a dose of 7.35 mM HP at 72 h of exposure was associated with a complete loss of green color in light microscopy (Figure 5E). In addition, the number of SYTOX Green-positive cells was high, while Chl and PC fluorescence decreased (Figure 5F). This observation was also in agreement with the macroscopic appearance of cultures shown in Figures 1B,C.

Light microscopy also showed subtle morphological alterations mainly within internal structures (Figure 5E).

**FIGURE 6:**
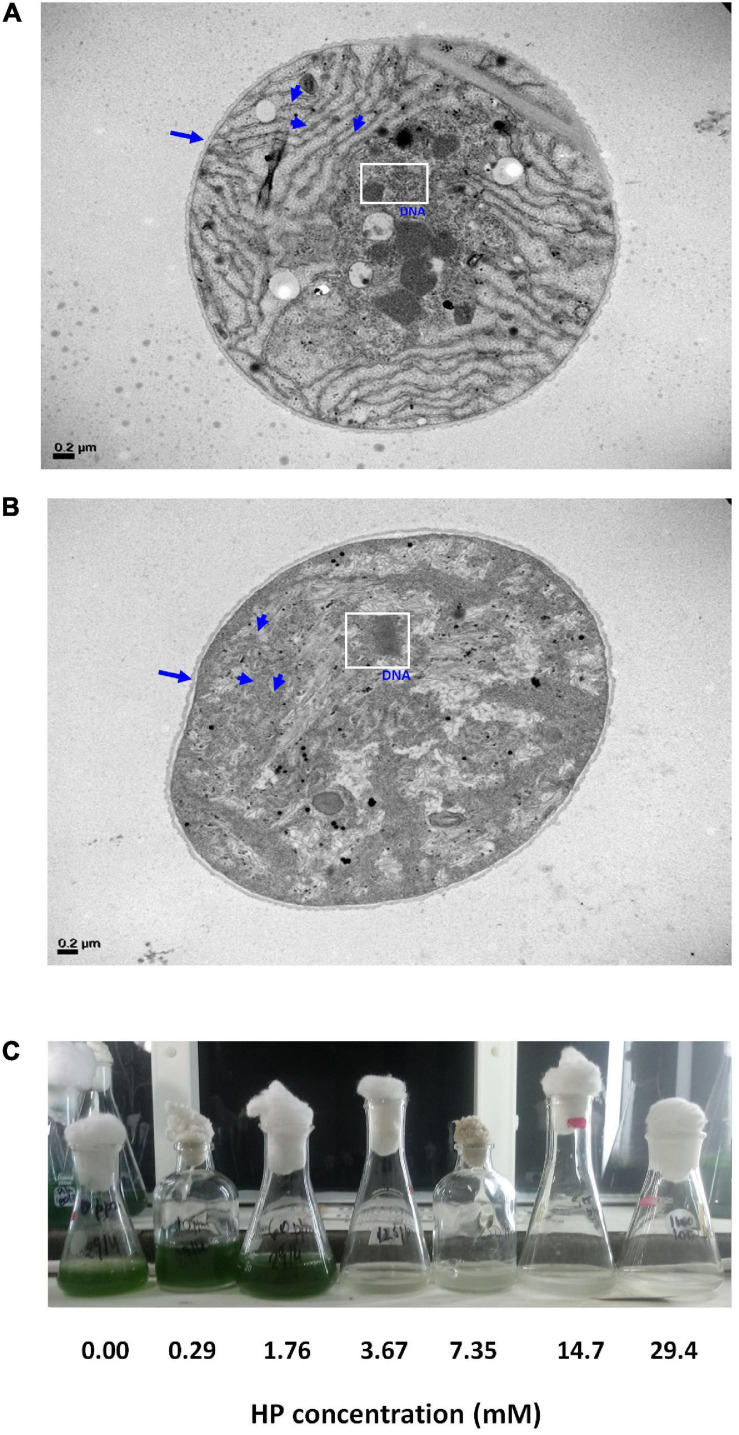
Electron microscopy of *M. aeruginosa* RCD and regrowth potential. **(A)** Untreated sample observed with TEM. Cell wall (arrow) and thylakoids (arrowheads) can be observed with its normal ultrastructure. The white square shows the normal appearance of DNA. **(B)** A sample treated with 4.26 mM HP at 72 h of exposure. Cell wall extensively disrupted (arrow) and thylakoids were disintegrated (arrowheads). The white square shows partial degradation and condensation of DNA. **(C)** *M. aeruginosa* culture after 15 days of being treated with HP.

Transmission electron microscopy (TEM) analyses showed that HP-treated at 72 h of exposure cells presented a less dense stroma, and distortion of the cell membrane. The thylakoids were partially disintegrated, and extensive cytoplasmic vacuolation was observed. At EC_50_ HP dose, cells showed highly condensed chromatin (Figures 6A,B). According to FSC signal, 3.67 mM of HP caused 10% shrinkage at 48 h (Figure 3A).

### ROS Formation and Measurement HP Decomposition Induced by HP

**FIGURE 7:**
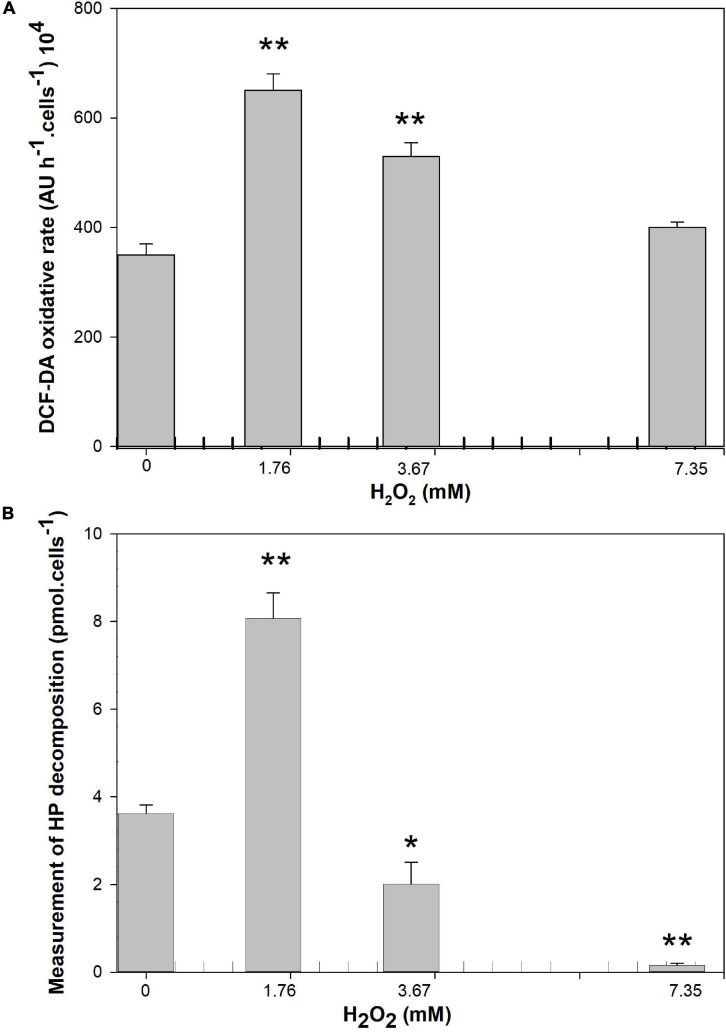
Effect of HP addition on **(A)** DCF-DA oxidation rate after 24 h of exposure (expressed as arbitrary units in 1 h exposure). **(B)** HP decomposition activity (Arbitrary Units per cell) after the exposure 24 h at 1.76, 3.67, and 7.35 mM of HP (*n* = 4). Each bar represents mean ± standard deviation (*n* = 4). Asterisk indicates the statistical difference relative to the control *(*p* < 0.05) and **(*p* < 0.01).

Changes in ROS and HP decomposition activity were measured in *M. aeruginosa* cultures after 24 h of exposure to HP (Figures 7A,B). Samples treated with 1.76 mM HP and 3.67 showed about two-times increase (*p* = 0.001) and 1.5-times increase (*p* = 0.008) in DCFH-DA compared to the control, while at 7.35 mM no significant differences with respect to the control were observed (*p* > 0.05) (Figure 7A).

A significant two-times increase in HP decomposition activity was observed with 1.76 mM HP (*p* = 0.001) compared to the control. At higher concentrations, the measurement of HP decomposition yielded lower values than in the control samples (1.8 and 24 times less than control with 3.67 mM and 7.35 mM HP respectively; *p* = 0.023 and *p* = 0.001 respectively) (Figure 7B).

### Regrowth Potential of Treated Samples

From the estimations obtained with cdf_dead(x) and pdf_dead(x) functions, it is clear that some cells can tolerate treatments at EC_50_. This fraction of resistant cells becomes progressively smaller with increasing HP concentrations. However, they can still have proliferative potential provided they have survived the HP treatment. Therefore, we evaluated the regrowth potential of samples after 15 days of being treated with HP as described in the materials and methods section (Figure 6C). Only HP doses up to 1.76 mM allowed growth, and higher doses produced cell death. No visual growth was observed in the culture after treatment with 3.67 mM. These doses suppressed *M. aeruginosa* regrowth. HP degradation in control samples (BG-11 medium) ranged between 10 and 15% after 72 h within the range of concentrations used in the assay (Table 1). HP disappeared from the batch cultures within 24–72 h after addition of 0.29 and 1.76 mM, while residuals values of HP were detected after addition of higher HP concentrations. At the end of the experiment (72 h), HP residual concentrations were < 0.05 mM with 0.29 and 1.76 mM HP treatment. At 3.67, 7.35, 14.70, and 29.40 mM treatment, HP residuals were 0.58, 2.44, 4.85, and 7.94 mM respectively (Table 1).

**TABLE 1:** Residual hydrogen peroxide concentration of *Microcystis aeruginosa* culture after addition of different HP concentrations.

Time (h)	HP residual (mM)					
0	0.29	1.76	3.67	7.35	14.70	29.40
1	<0.05	0.12	1.90	4.95	11.14	22.52
24	<0.05	0.08	1.50	3.03	6.85	9.50
48	<0.05	0.05	0.88	2.79	5.08	8.67
72	<0.05	<0.05	0.58	2.44	4.85	7.94

Cells were not able to initiate growth in the presence of HP residual concentrations (Figure 6C), and the latter could be a potential cause of the observed result. Assays showing no re-growth confirmed the occurrence of cell death.

This reveals that EC_50_ (3.77 and 4.26 mM at 48 and 72 h, respectively) may represent a point of no return and commitment to the process of cell death (Figure 6C).

### Toxin Content

The initial level of [D-Leu^1^]MC-LR in the culture of *M. aeruginosa* was 92 ìg.L^–1^, and achieved 178 ìg.L^–1^at 72 h. By contrast, the concentration of [D-Leu^1^]MC-LR in cultures treated with 4.26 mM HP (EC_50_) for 72 h was 70ìg.L^–1^, representing 60% less than the production in the control sample. The values of live cells were 9.6 10^5^ and 1.5 10^6^ cell.mL^–1^ for control samples at 0 and 72 h, respectively. The number of live cells after treatment with 4.26 mM HP (EC_50_) for 72 h was 8.3 10^5^cell.mL^–1^. The MC cell quota (Q_*MC*_) was calculated as the quotient of [D-Leu^1^]MC-LR and the number of live cells. The Q_*MC*_ of the control increased from 95 to 118 fg⋅cell^–1^ at 0 and 72 h, respectively. Q_*MC*_ of cells treated with 4.26 mM HP at 72 h was 84 fg⋅cell^–1^, therefore decreasing 29% compared to the control level and confirming the inhibition of [D-Leu^1^]MC-LR biosynthesis. These results indicated that after 72 h of EC_50_ treatment (4.26 mM HP), half of the population had die, the cell membrane was degraded, and the toxin synthesis was inhibited.

## Discussion

The concentrations used in the present work were not intended for environmental applications such as cyanobacterial bloom control but to determine the range within which RCD most probably occurs. Nevertheless, the still limited number of field studies examining cell death in either marine or freshwater systems severely limits the understanding of the environmental conditions that elicit RCD in cyanobacteria (Aguilera et al., 2021).

The activation of cell death pathways in response to stressors like HP is considered RCD, regardless the particular genetic program actually involved and the morphological and biochemical features observed (Galluzzi et al., 2018). The occurrence of RCD in a cell population exposed to a stressful agents such as HP is not synchronous but is rather a stochastic process. At a given time, different cells will be at different stages of the RCD process. This shows the importance of a precise definition of the criteria used to assess a dying population (Kroemer et al., 2005).

In this study, we have evaluated RCD induced by HP in a culture of *M aeruginosa* over a broad range of concentrations at 48 and 72 h. We obtained a characterization of RCD by means of two probabilistic functions.

The first function (cdf_dead) is a probability function that we obtained by non-linear regression to estimate the cumulative probability of death as a function of HP concentration. The second function (pdf_dead) is the derivative of the first function and represents the theoretical exact concentration of HP required to elicit cell death by different cohorts of cells within the population. The parameters of these two functions are intrinsically linked to RCD, and to the equilibrium between cellular pathways in favor and against RCD. The modeling functions were based on the concept of binary outcome “dead or alive” cells in order to benefit from the ability of flow cytometry to screen thousands of individual cells, scoring several fluorescent and dispersed light parameters. We were thus able to characterize the variability of *M. aeruginosa* population regarding their susceptibility to HP-induced RCD. The cdf_dead function showed a sigmoidal shape with and EC_50_ of 3.77 mM HP at 48 h and 4.26 mM HP at 72 h.

The pdf_dead function showed that EC_50_ was also the most frequent HP concentration that induced RCD. The shape of this function identified the frequency of resistant cells that required more than EC_50_ at the right tail, and the frequency of susceptible cells that required less than EC_50_ to initiate RCD at the left tail. The skewed shape with long right tail observed at 48 h, indicated a greater abundance of resistant *M. aeruginosa* cohorts in the culture, as compared to the right tail of the function obtained at 72 h.

A distinctive characteristic of stress-induced RCD is the restraining force of cell survival pathways. However, the stochastic attribute of these cellular processes has been rather neglected. Stress-induced RCD does not occur at the same dose in every cell, but instead it is observed throughout a whole dose range with variable figures.

An immediate conclusion from the cdf_dead function is that the HP dose required for the occurrence of RCD was variable among the cells in the cyanobacteria population. There was individual variability and some cells were more resistant to HP than others. Therefore, an incremental fraction of dead cells as a function of HP concentration indicated the amount of damage required to overcome resistant pathways, and trigger RCD in the different cohorts of cells in the population.

An important aspect of the cdf_dead function that has a relevant biological meaning is that EC_50_ is not only the HP concentration that is expected to kill 50% of cells, but is also the minimal dose required to achieve that figure. To demonstrate this concept, we could suppose that a concentration x was the EC_50_ and was not minimal; then there would be a lower concentration y that kills 50% of the population and x would not be the EC50, which is a false conclusion (*reduction ad absurdum*). Therefore, since EC_50_ is the minimal dose initiating cell death in 50% of cells, it is also by definition a measure of RCD.

In addition, the pdf_dead function allows determining the frequency of cells in the population that requires a given exact dose of HP to initiate RCD, something that is not possible with cdf_dead cumulative function. Thus, skewed distributions can contribute to identify increase abundance of susceptible or resistant cohorts within the cyanobacteria population. The theoretical value of pdf_dead function relies in that it provides information that is not possible to obtain experimentally in a straightforward manner. Anytime a concentration of HP causes death in 50% of cells, it would kill those cells requiring exactly EC_50_ dose, but also cells requiring any dose below EC_50_. In other words, there is no experimental method of tracking a single cell with incremental exposure to HP to identify the exact dose required to elicit cell death. Therefore, the pdf_dead function anticipated that EC_50_ is the most frequent exact HP dose eliciting RCD, and is the minimal dose required to cause death in 50% of the population. In addition, the pdf_dead function supports the notion that the mode of cell death at EC_50_ is RCD, regardless of the genetic pathways involved in its active execution. The frequency of RCD cell death becomes rapidly lower at very high HP doses. Above the EC_100_ HP dose, RCD is highly unlikely, therefore supporting the observation that passive cell death (ACD) occurs at very high HP doses, due to massive cellular destruction and impairment of metabolic support for active cell death.

Visualization and clustering algorithms confirmed that the change of the five-dimensional phenotype from live clusters to dead clusters was initiated at HP doses close to EC_50_ (iRCD metacluster in SPADE graph). The cell phenotype transitioned smoothly to the late-dead metacluster at high HP doses where RCD is fully accomplished according to the pdf_dead and cdf_dead functions.

The activities of antioxidant systems increase at relatively low environmental stress, and decrease at increased stress because of irreversible damage to algal cells (Almeida et al., 2017). It can be seen that at 1.76 mM the ROS and HP decomposition activity increased significantly. This hormetic effect (characterized by low dose stimulation and high dose inhibition) probably reflects the intracellular signaling role of HP, triggering increased HP decomposition activity at low doses as anti-oxidant stress responses. Treatment with low doses of HP (1.76 mM), caused a rapid increase in ROS inside the cells that generates the antioxidant response (increase in HP decomposition activity), which in turn allowed inhibiting cell death path and a regrowth, as observed after 15 days.

Cells exposed to EC_50_ HP showed that RCD overcomes the defenses that maintain the antioxidant balance (HP decomposition activity decreased significantly). Both transmitted light microscopy and TEM confirmed flow cytometry data, showing morphology often seen in RCD such as increased distortion of cell membrane, highly condensed chromatin, partial disintegration of thylakoids and extensive cytoplasmic vacuolation. These results suggest that RCD features occurred at EC_50_ HP concentrations.

Redox alterations have been proposed as a potential trigger of RCD. The oxidative potential of the intracellular medium not only inhibits several enzymatic activities, but also causes direct structural damage to organelles and membranes (Galluzzi et al., 2015). The mechanism of HP as algaecide is considered to involve reactive hydroxyl radicals produced by HP that damage cells by the oxidation of lipids, proteins and DNA, eventually leading to severe oxidative stress (Apel and Hirt, 2004; Latifi et al., 2009).

RCD can be activated once adaptive responses to perturbations of the intracellular microenvironment are overcome. At EC_50_ at 48 and 72 h (3.77 and 4.26 mM) a high proportion of cells had started to progress along a non-return path. This was confirmed morphologically by direct structural damage to cell membrane (Figures 5, 6), biochemically by high ROS production (Figure 7A), low antioxidant defense by HP decomposition (Figure 7B), and macroscopically by lack of re-growth after 15 days (Figure 6C). The evidence of these destructive processes during RCD at EC_50_ should be linked to the finding that inhibition of [D-Leu^1^]-MC-LR biosynthesis up to 60% compared to control value was also confirmed at EC_50_. These results are in line with those reported by Zhou et al. (2020), where HP treatment for 7 days with a dose range 0.26–0.44 mM caused a total MC-LR reduction of 82–86% compared to control. This finding raises the possibility that cyanobacterial toxin could be consistently degraded in cells undergoing RCD, although not necessarily in uncontrolled ACD at high HP doses (Zhou et al., 2020).

Several questions remain to be answered regarding the peculiarities of RCD phenotype and the molecular machinery involved in RCD in *M aeruginosa*. It is still necessary to characterize the RCD pathways by genetic, biochemical and pharmacologic approaches, and to identify the signaling effectors. The latter includes membrane ligands, adapters, second messengers, and executioners of the presumably different RCD pathways. Modulating specific effectors of different RCD pathways in Cyanobacteria could be the main objective to trigger their death, and consequently control harmful algal blossoms without affecting other organisms.

The EC_50_ and the stochastic functions presented in this study allows to identify which doses of the stressor cause RCD. This, together with the multidimensional transitional phenotypic analysis of single cells contribute to the characterization of cyanobacterial cell death pathways. Our N-dimensional approach can be expanded and include additional parameters (e.g., caspase activity, annexin binding, and other protease activities) and even applied to single cell RNAseq data. Transitional maps to trace gene expression changes through RCD can be created with hundreds of gene expression parameters and flow cytometry data using more specific algorithms such as Wanderlust and Monocle (Saelens et al., 2019). Moreover, for field approaches where RCD is expected to be triggered by multiple environmental factors, EC_50_ and the stochastic functions could be a practical, and fast monitoring tool. For example, flow cytometry has been recently used to characterize the profile of cyanobacteria species in water samples from 36 water sites in New Jersey, USA, and even proposed as an efficient real time monitoring method (Patel et al., 2019). An extension of this kind of profiling studies could be the assessment of EC50 after increasing HP doses, and therefore estimating the HP doses required to trigger RCD in individual cyanobacteria, by conducting incubation experiments using environmental samples from water bodies (Dugenne et al., 2015; Lusty and Gobler, 2020).

## Conclusion

Our findings showed that EC_50_ was associated with the initiation of RCD showing a progressive transition of a five-dimensional phenotype toward a fully accomplished RCD. This phenotype included decreased chlorophyll, phycocyanin, decreased cell size and cellular complexity, cell membrane damage, and was associated with morphological signs of intracellular disruption, vacuolation, and condensation of DNA at EC_50_. The stochastic functions pdf_dead and cdf_dead provided the HP dose ranges within which RCD was induced in *M aeruginosa*, and above which minor resistant cohorts had no regrowth potential. Inhibition of [D-Leu^1^]-MC-LR biosynthesis up to 60% compared to control value was also confirmed at EC_50_.

## Data Availability Statement

The raw data supporting the conclusions of this article will be made available by the authors, without undue reservation.

## Author Contributions

LG and GB conceptualized the study. TL, IJ, and AA contributed with experimental assay. All authors wrote, reviewed, and edited the manuscript.

## Conflict of Interest

The authors declare that the research was conducted in the absence of any commercial or financial relationships that could be construed as a potential conflict of interest.

## Publisher’s Note

All claims expressed in this article are solely those of the authors and do not necessarily represent those of their affiliated organizations, or those of the publisher, the editors and the reviewers. Any product that may be evaluated in this article, or claim that may be made by its manufacturer, is not guaranteed or endorsed by the publisher.

## Footnotes

1
http://pengqiu.gatech.edu/software/SPADE
