# Supplementary material for: A Stochastic Characterization of Hydrogen Peroxide-Induced Regulated Cell Death in Microcystis aeruginosa
Source: Front Microbiol. 2021 Jul 28;12:636157. doi: 10.3389/fmicb.2021.636157 (PMC8356053; doi:10.3389/fmicb.2021.636157)
Supplement: Supplementary file 1 [file Data_Sheet_1.PDF]

Supplemental figure 1:

Visualization of cells in the five-dimensional space through the UMAP algorithm

A) Transitional phenotypes of cells treated with HP, visualized through single parameter values of cells plotted as heatmaps in UMAP bidimensional graphs. The dotted arrow indicates the transition of the five-dimensional phenotype as HP was increased, while the color code indicates single parameter value of individual cells in arbitrary units.

B) Cells from individual samples treated with HP, visualized in UMAP plots to track phenotypic transition of cells, according to the HP treatment they received. The lower right panel is a reference plot with pooled samples. Color code corresponds to frequency of cells.

Supplemental figure 2:

Visualization of cells in the five-dimensional space through the t-SNE algorithm

A) Transitional phenotypes of cells treated with HP (pooled samples), visualized through single parameter values of cells plotted as heatmaps in t-SNE bidimensional graphs. The dotted arrow indicates the transition of the five-dimensional phenotype as HP was increased, while the color code indicates the single parameter value of individual cells in arbitrary units.

B) Cells from individual samples treated with HP, visualized in t-SNE plots to track phenotypic transition of cells according to the HP treatment they received. The lower right panel is a reference plot with pooled samples. Color code corresponds to frequency of cells.

Supplemental figure 3:

Definition of five metaclusters of cells considering single parameter average values in 100 nodes of SPADE tree graphs

Tree-like SPADE graphs made of 100 nodes grouped by phenotypic similarity were explored for distribution of each single parameter. Color code represents average level of

single parameters at each node. Transitions in all parameters among nodes were used to track phenotypic changes caused by HP treatment, and define five main metaclusters (as indicated in legends): two of live cells and three of dead cells. We denoted the metacluster iRCD as initial phenotype of regulated cell death. Transitions and identification of dead cells were in agreement with conventional flow cytometry manual analysis through sequential univariate and bivariate plots.

Supplemental figure 4:

Unsupervised classification of cells through a self-organized network algorithm (flowSOM) with 144 nodes and eight automatically generated metaclusters.

Tree-like flowSOM graph made of 144 nodes grouped by phenotypic similarity. The flowSOM algorithms used a 12 by 12 lattice (known as a Kohonen map) and a neural network approach, to group cells into nodes of phenotypically very similar cells considering the five parameters. The size of each node is proportional to the amount of cells grouped together. The color-coded pie chart shows the average level of the five parameters in each node. The distance between any two nodes along the path is proportional to their phenotypic dissimilarity. Metaclusters number 0 to 7 were automatically defined by flowSOM algorithm through a hierarchical clustering step, and are represented by a background color as indicated in the legend. The algorithm identified two big metaclusters of live and dead cells that can be recognized by the abundance of nodes with high PC, Chp, SSC with low SYTOX Green (Live), and low PC, Chp, and SSC with higher SYTOX Green values (Dead). We denotes iRCD the small metacluster with a transitional phenotype between live and dead cells (arrow), where we recognized the initial phenotype of regulated cell death. Transitions and identification of dead cells were in agreement with conventional flow cytometry manual analysis through sequential univariate and bivariate plots.

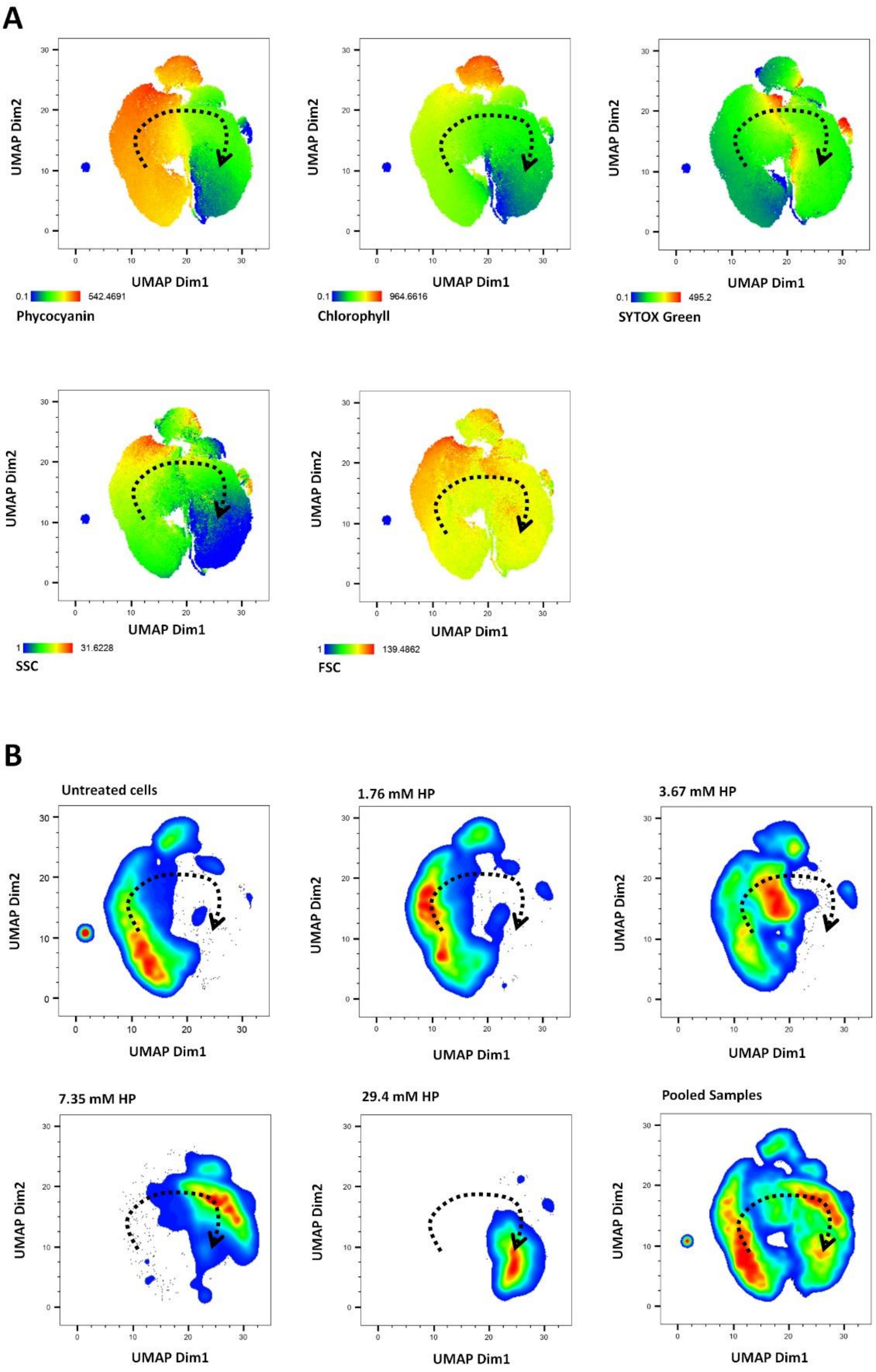

Supplemental figure 1:

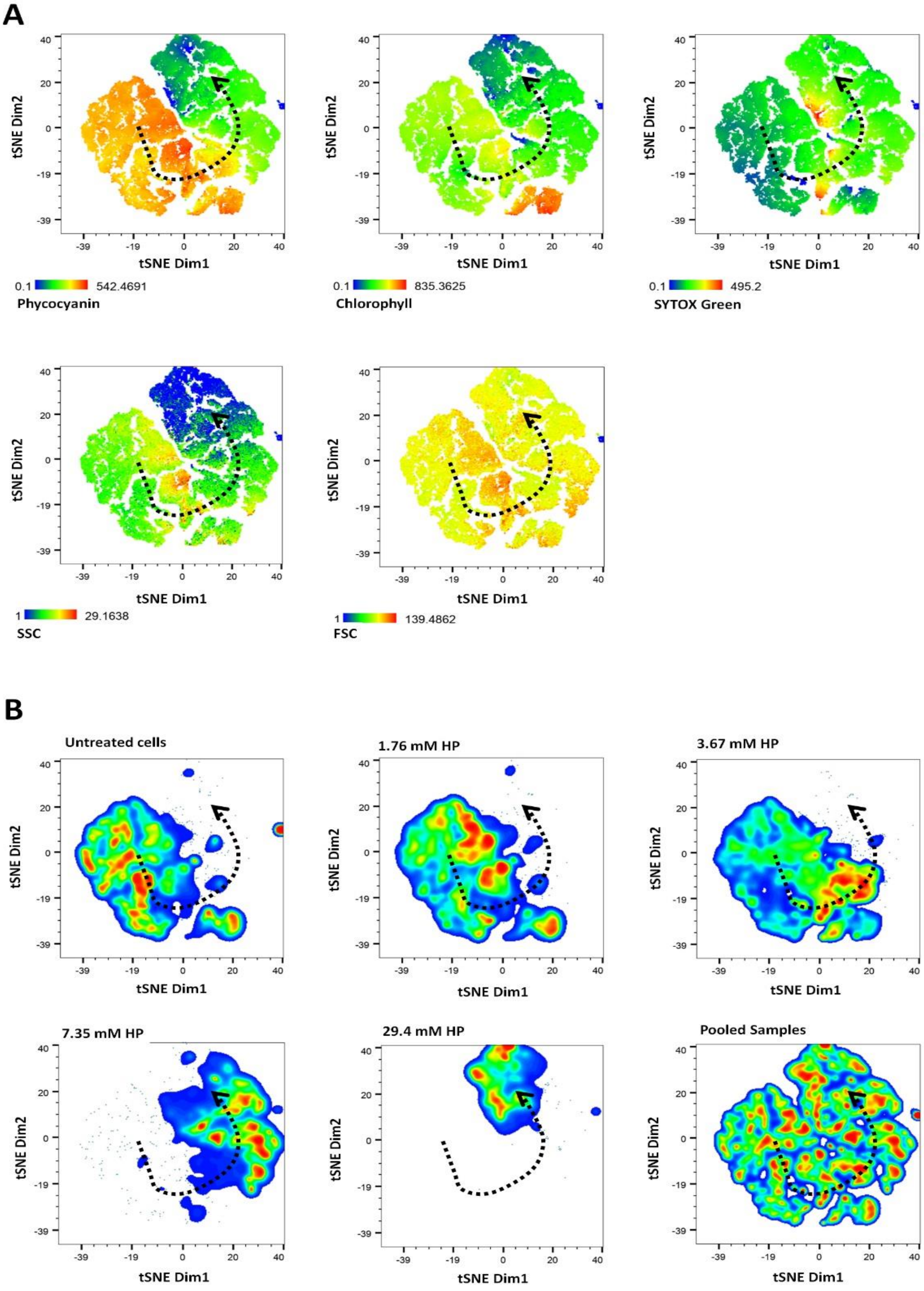

Supplemental figure 2

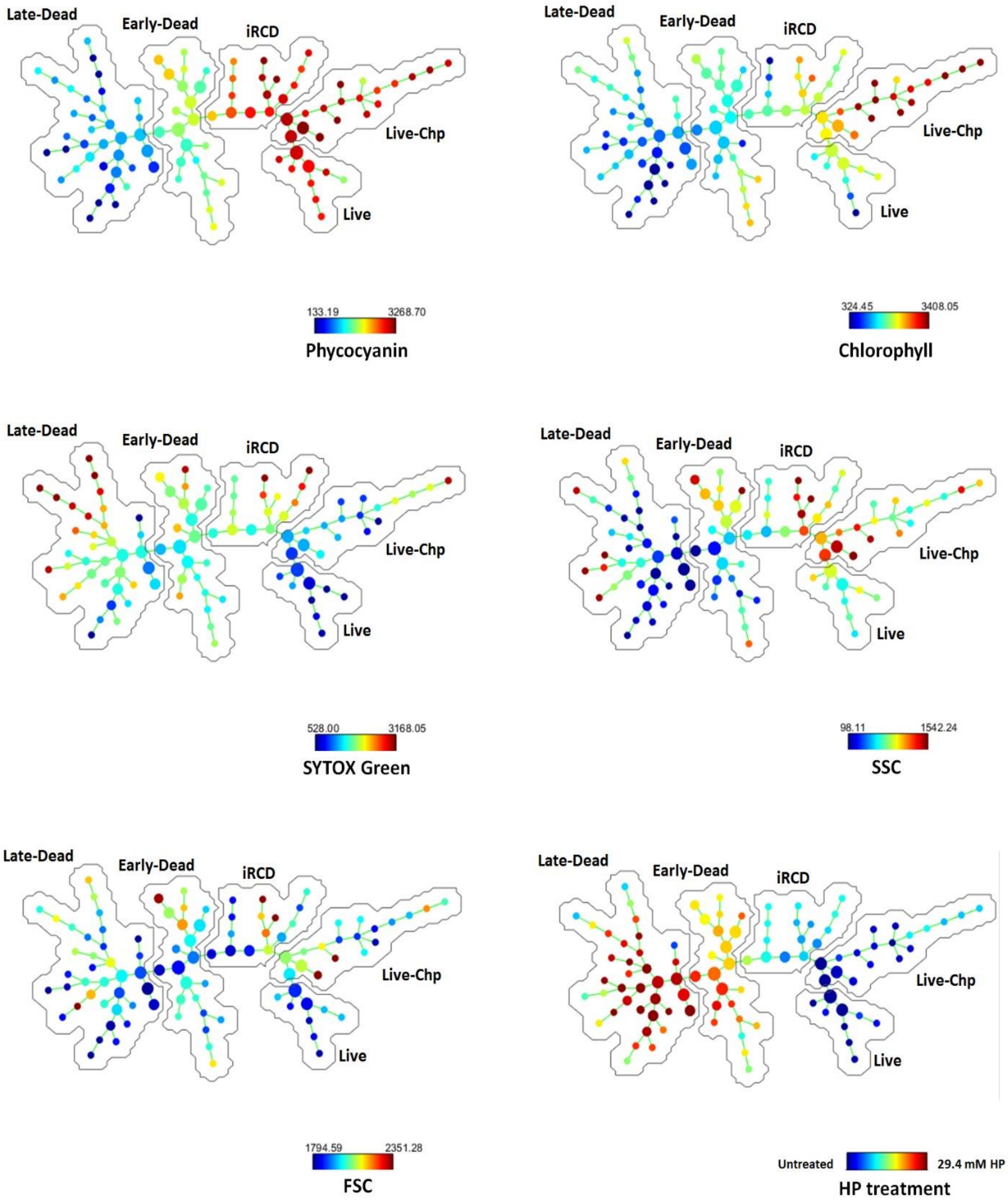

Supplemental figure 3

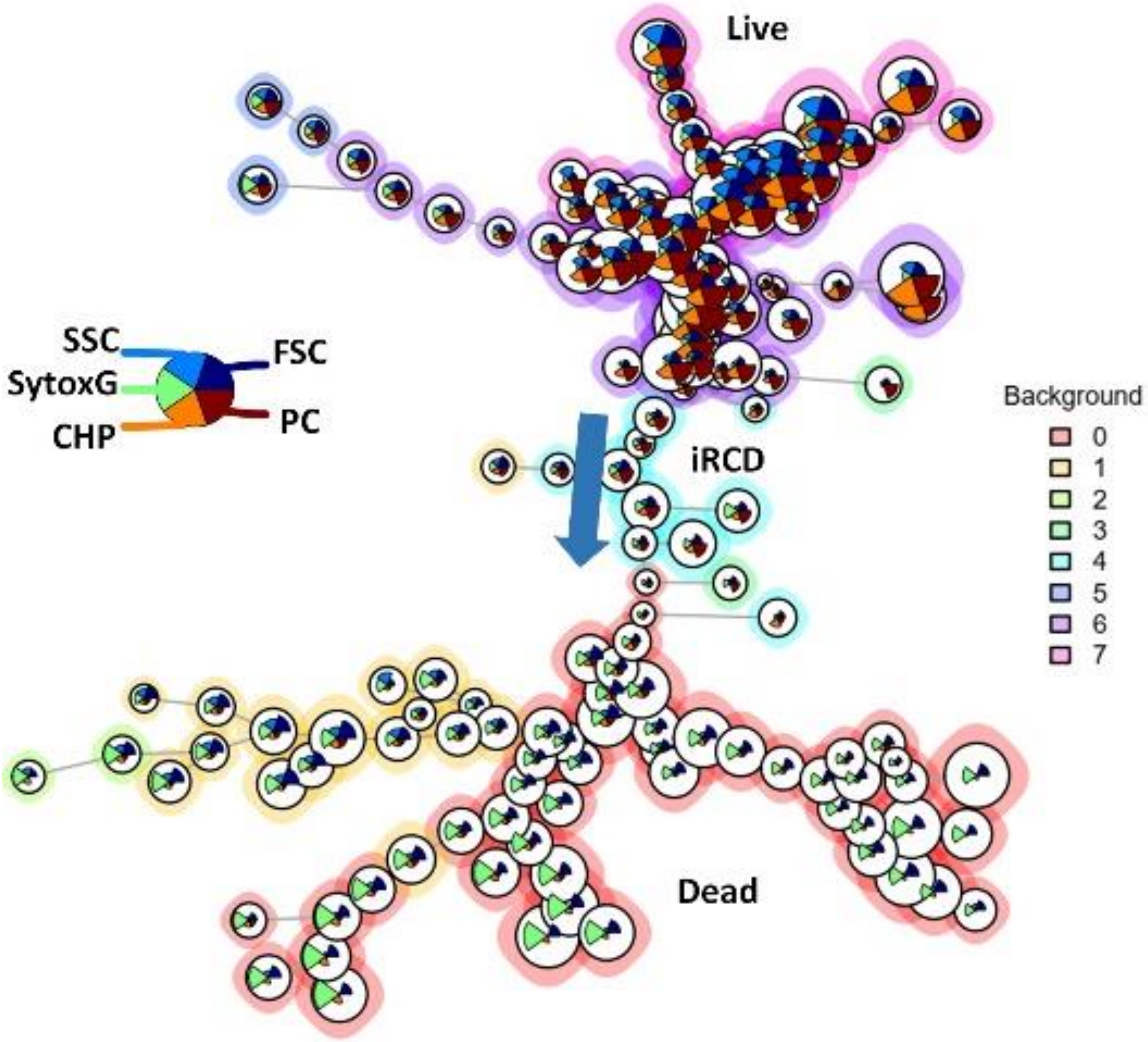

Supplemental figure 4
